# Freezing or death feigning? Beetles selected for long death feigning showed different tactics against different predators

**DOI:** 10.1002/ece3.8533

**Published:** 2022-02-07

**Authors:** Masaya Asakura, Kentarou Matsumura, Ryo Ishihara, Takahisa Miyatake

**Affiliations:** ^1^ Graduate School of Environmental and Life Science Okayama University Okayama Japan; ^2^ Department of Agriculture Kagawa University Kagawa Japan

**Keywords:** animal hypnosis, death feigning, freezing, predation, thanatosis, tonic immobility

## Abstract

Prey evolve antipredator strategies against multiple enemies in nature. We examined how a prey species adopts different predation avoidance tactics against pursuit or sit‐and‐wait predators. As prey, we used three strains of *Tribolium* beetles artificially selected for short (short strain) or long (long strain) duration of death feigning, and a stock culture (base population). Death feigning is known to be effective for evading a jumping spider in the case of the long strains, while the present study showed that the long‐strain beetles used freezing against a sit‐and‐wait type predator, *Amphibolus venator*, in this study. The short‐ strain beetles were more easily oriented toward predators. The time to predation was also shorter in the short strains compared to the long strains. The results showed that, as prey, the short strains displayed the same behavior, escaping, against both types of predators. Traditionally, death feigning has been thought to be the last resort in a series of antipredator avoidance behaviors. However, our results showed that freezing and death feigning were not parts of a series of behaviors, but independent strategies against different predators, at least for long‐strain beetles. We also examined the relationship between a predator's starvation level and its predatory behavior. In addition, the orientation behavior toward and predation rate on the prey were observed to determine how often the predatory insect attacked the beetles.

## Graphical abstract

Prey evolve anti‐predator strategies against multiple enemies in nature. We examined how a prey species adopts different predation avoidance tactics against pursuit or sit‐and‐wait predators. Traditionally, death feigning has been thought to be the last resort in a series of anti‐predator avoidance behaviors. However, our results showed that freezing and death feigning were not parts of a series of behaviors, but independent strategies against different predators, at least for long‐strain beetles. This result suggests a reason why preys show adaptive behavior to many kinds of natural enemies in nature.

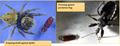

## 1 INTRODUCTION

Predation is a key agent of natural selection in prey species (Edmunds, 1974; Ruxton et al., 2018). To survive in the real world with multiple predators, animals evolve different defense capabilities that vary in their nature and efficacy in relation to predator sensory abilities and attack tactics (Hoverman & Relyea, 2007; Rojas et al., 2021).

Freezing and death feigning in insects have been considered as part of a series of tactics within a single antipredator behavior (Humphreys & Ruxton, 2018; Sakai, 2021). Traditionally, freezing is the first behavior a prey will exhibit when an enemy attacks, while death feigning has been thought to be the last resort in antipredator avoidance behaviors. It is believed that when an insect senses the presence of an enemy, it will first freeze, and if the enemy does not give up, it will feign death as a last resort (Honma, 2021; Sakai, 2021).

Death feigning means becoming immobile on one's back in many cases, and is a behavior that has recently received a lot of attention (Humphreys & Ruxton, 2018; Franks et al., 2020; Sendova‐Franks et al., 2020; Sakai, 2021). On the other hand, freezing is the act of remaining in the same position (i.e., not turning over on one's back) and not moving in front of the predator when a predator threat is imminent. Freezing has been identified as an alternative to the antipredator strategy of immobility (Hennig, 1979; Sakai, 2021). For example, freezing is observed in the Colorado potato beetle. Freezing is sometimes called “quiescence,” as in a study by Acheamgpong and Mitchell (1997).

In this study, we hypothesized that death feigning and freezing are not tactics in a series of behaviors, but rather function as different defenses against different enemies. If this is correct, the two behaviors can be better put into the context of proximate causes of a behavioral switch (i.e., to clarify the underlying pleiotropy between death feigning and freezing that is mediated by dopamine).

As a model system for examining the evolution of death feigning and freezing, a series of studies have been conducted using *Tribolium* beetles as prey and a jumping spider, *Hasarius adansoni*, as predators (Miyatake et al., 2004, 2009). In these studies, strains artificially selected for shorter (short strain) and longer (long strain) durations of death feigning have been established. The jumping spider attacks a beetle, but the beetle's body is so hard that the spider releases it. A beetle of the short strain moves in the sight of the spider, so the spider attacks it two or three times and then eats it. However, when an adult beetle of the long strain is attacked, it stops moving and feigns death, so the spider watches its prey for a while but then loses interest (Miyatake et al., 2004), or if there is another moving prey nearby, the spider's interest would shift to it, and the beetle that feigned death survives (Miyatake et al., 2009; see video in Matsumura, Iwaya, et al., 2021; Matsumura, Yumise, et al., 2021 for another beetle: *Gnatocerus cornutus*).

On the other hand, the rates of being preyed on of the long and short strains of *Tribolium freemani* selected for diverse durations of death feigning were also compared, and the beetles derived from short strains were preyed on more frequently than those of the long strains when the predator used was a sit‐and‐wait type, that is, *Amphibolus venator* (Konishi et al., 2020).

However, to date, we have not observed in detail the behavior of the prey when *Tribolium* beetles were attacked by *Amphibolus venator*. Also, the predatory behavior of Assassin bugs has not been well studied (but see Wignall et al., 2011; Wignall & Taylor, 2011). Therefore, we examined the predator‐avoidance behaviors of long and short strains of *Tribolium castaneum* and *T*. *confusum* against *Amphibolus venator* in the present study. We found for the first time that *Tribolium* beetles adopt different strategies, freezing or death feigning, against different species predators.

We also examined the relationship between a predator's starvation level and predatory behavior. In addition, the orientation behavior and predation rate on the prey were observed to determine how often the predatory insect attacks the beetles.

## 2 MATERIALS AND METHODS

### 2.1 Insects

The population of *Amphibolus venator* used as predators was collected on the main island of Okinawa (Urasoe City) in May 2015 and has been reared for generations at Okayama University since then. This population was reared in circular plastic Petri dishes with a diameter of 30 mm and a height of 10 mm in an incubator maintained in 16L8D hours light and dark and a temperature of 29°C. The base population of *T*. *castaneum* (see Miyatake et al., 2004) was used as bait. Three *T*. *castaneum* beetles or larvae were fed to each *A*. *venator* individual once a week.

Two *Tribolium* beetle species, *T*. *castaneum* and *T*. *confusum*, were used for predation experiments. They were reared in an incubator controlled at 25°C for *T*. *castaneum* and 27°C for *T*. *confusum* and 16L8D hours. The beetles were fed graham flour containing 5% yeast as food. A stock culture (=base population) reared over 20 years in the laboratory and short and long (death‐feigning) strains (see Miyatake et al., 2004) were used for the experiments. Death‐feigning behavior was induced by touching the abdomen of the beetle three times with a wooden stick. When the beetle feigned death, the duration was recorded for both species. If the beetle was unresponsive to all stimulations, it was recorded as a non‐responsive individual. The protocol for artificial selection for the duration of death feigning was described in Miyatake et al. (2004) for *T*. *castaneum*, and in Nakayama et al. (2010) for *T*. *confusum*. Briefly, 100 males and 100 females were randomly selected from the base stock culture, and their death‐feigning behavior was observed (F0 generation). The males and females (ten each) with the shortest duration of death feigning were selected to propagate the short strain; similarly, the ten males and ten females with the longest duration were selected to propagate the long strains. The males and females of each strain were placed in a small plastic cup with medium and allowed to copulate and lay eggs for 1 week. Pupae arising from the eggs were stored as separate‐sex groups in different plastic cups and allowed to emerge. When the adults reached 10–15 days of age, 100 males and 100 females were randomly selected from each strain, and their death feigning was observed again (F1 generation). Two and three selection replicates for *T*. *castaneum* and *T*. *confusum*, respectively, were established for the short and long strains in this manner and subsequently maintained in the chamber under the same conditions. This artificial selection was repeated for more than 29 generations in *T*. *castaneum* (Matsumura & Miyatake, 2018) and more than 27 generations in *T*. *confusum* (Nakayama et al., 2010). The beetles were used in the experiments 2–4 weeks after hatching.

### 2.2 Experimental methods

#### 2.2.1 Experiment 1: Freezing behavior

**FIGURE 1:**
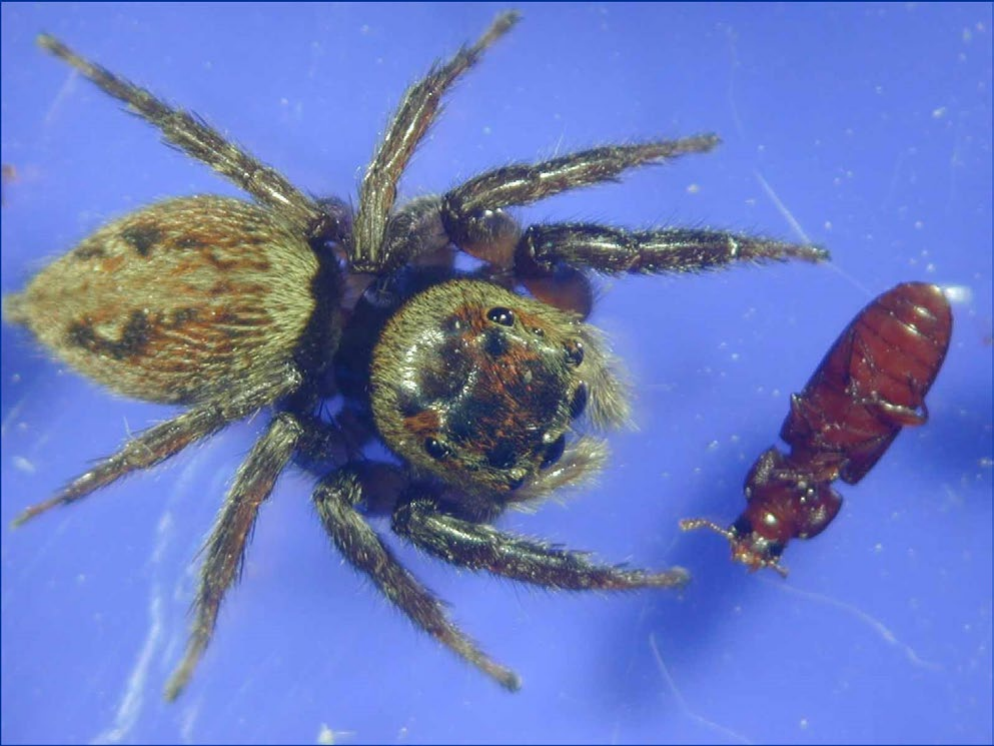
A *Tribolium castaneum* beetle feigning death in front of *Hasarius adansoni*

**FIGURE 2:**
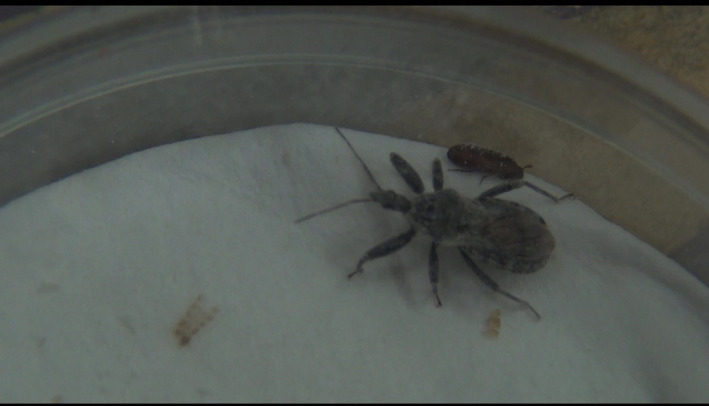
A *Tribolium castaneum* beetle freezing at side of *Amphibolus venator*

We examined whether *Tribolium* beetles feigned death or froze when attacked by *A*. *venator*. Death‐feigning strains (short and long strains) of both *T*. *castaneum* and *T*. *confusum* were used. The experiments were conducted in plastic Petri dishes with a diameter of 30 mm and a height of 10 mm. The bottom of the Petri dish was covered with a sheet of filter paper, and a folded sheet of filter paper was placed in the center as partition to separate the test insects. Five beetles and one predatory bug were used at the same time for observation of the long strain because the long strain hardly moves, and thus the two species rarely meet each other (predator and preys), and one beetle and one predatory bug were put in for observation of antipredator behavior of short strains against an *A*. *venator* adult. After 5 min, the partition was removed, and they were brought face to face. Video recordings were made with a video camera for 2 h, and the behavior was analyzed. In this experiment, we used *A*. *venator* starved for 7 days. When attacked by *A*. *venator*, the prey would either feign death (Figure 1: the case of *A*. *hasarius*, also see Miyatake et al., 2004) or freeze (Figure 2: *T*. *castaneum* remained in the same position, that is, not turning over on its back, and just becoming immobile when a predator threat was imminent; also see Video 1 and 2). Long (*N* = 44) and short (*N* = 42) strains of *T*. *castaneum*, and long (*N* = 21) and short (*N* = 20) strains of *T*. *confusum* were used in the experiment. Sample sizes for all experiments are listed in Table S1. We used the replicated lines “A and B” of *T*. *castaneum* (long or short strains: see Miyatake et al., 2004) and the replicated line “A” of *T*. *confusum* (long or short strains: see Nakayama et al., 2010). The experiment was conducted between 1:00 pm and 6:00 pm in a room at a temperature of 25°C.

**VIDEO 1:** Responses of *Tribolium castaneum* to attacking by *Amphibolus venator*. Predator chases their prey by sensing the vibrations of walking beetles. The beetle freezes when it senses the approach of a predator. When the beetle becomes freeze, the predator loses sight of the beetle

**VIDEO 2:** Responses of *Amphibolus venator* to a freezing *Tribolium castaneum*. If the beetle remains immobile for a while, the predator loses interest to the beetle and leaves the area

#### 2.2.2 Experiment 2: Effect of starvation period and orientation toward predator

The experiments were conducted in plastic Petri dishes as in the experiment 1. The stock culture (=base population), and short and long strains were used in the experiment. One predatory bug was placed on one side of the Petri dish and one beetle on the other. After 5 min, the barrier was removed, and the two were brought face to face. We observed each behavior of the two insects when they were in contact with each other while measuring the time with a stopwatch. Observations were made until *A*. *venator* attacked the prey, and if it did not attack for more than 2 h after removal of the partition, it was recorded as "no predation". We defined "orientation" as a change in the direction of its body toward the prey's location. When the predator pinned the prey between its legs and blocked the prey's movement, it was considered "predation". Whenever this behavior occurred, the predator ate the prey.

The presence or absence of orientation, predation, time to orientation, and time to predation for each sex were recorded by a video camera. Later, we analyzed the four measures. All predators examined oriented toward the prey before predation. Predatory bugs were used in the experiments after 1 day (1d), seven days (7d), and fourteen days (14d) of starvation after we confirmed that the bug had eaten at least one of three prey beetles we gave the bug (= three levels of starvation). The numbers of *T*. *castaneum* used in the experiment (sample size) were 55 for 1 d, 78 for 7 d, and 5 for 14 d of starvation, respectively.

We examined the orientation behavior of predators toward the long and short strains and their predation rates when the bait was *T*. *castaneum* and *T*. *confusum*, respectively. All experiments were conducted under the same conditions as in the experiment 1. Thirty‐one *T*. *castaneum* individuals of the long strain were used in the experiment using the long strain and 20 individuals of the short strain when the predator was starved (7d), and 17 individuals of the long strain and 27 individuals of the short strain were used when the predator was not starved (1d). The number of *T*. *confusum* used in the experiment was 180 individuals of the long strain and 194 individuals of the short strain. In the case of *T*. *confusum*, only starved predators (7d) were used for the experiment. We used the replicated line “A” of *T*. *castaneum* (long or short strain: see Miyatake et al., 2004) and three replicated lines (A, B, and C) of *T*. *confusum* (long or short strain: see Nakayama et al., 2010), which were selected by their duration of death feigning. Sample sizes for all experiments are listed in Table S1.

### 2.3 Statistical analysis

In analysis of experimental data focusing on the freezing behavior of *T*. *castaneum* and *T*. *confusum* from death‐feigning strains, it is important to include both beetle responses and predator responses in the analysis of foraging behavior of *A*. *venator* because predation occurred after the beetle responded (froze or did not freeze). Therefore, we first used an item response tree model (López‐Sepulcre et al., 2015) to analyze the foraging behavior by *A*. *venator*. In this analysis, each event is considered a "node", with beetle responses as node 1 and predator responses as node 2. The presence or absence of each event was analyzed as binary data with a binomial distribution (López‐Sepulcre et al., 2015). In this analysis, death‐feigning strains were used as a fixed effect, and predator ID and replicate lines of beetle strains (only *T*. *castaneum*) were used as random effects. We also tested freeze and predation rates separately in a logistic regression analysis as a post‐hoc test of the item response tree models. In this analysis, death‐feigning strains and sex (only *T*. *castaneum*) were used as a fixed effect, and replicate lines of death feigning strains as a random effect (only *T*. *castaneum*).

The data of experiments with the beetles from the stock culture also analyzed for time to orientation and for time to predation using the generalized linear mixed model assuming a gamma distribution. The statistical models for latencies of orientation and predation adopted in this analysis referenced AIC values (Table S2).

Because assassin bugs preyed after they orientated toward beetles, it is important to include both orientation and predation in the analysis of foraging behavior of *A*. *venator*. Therefore, we first similarly used the item response tree model (López‐Sepulcre et al., 2015) to analyze the foraging behavior by *A*. *venator*. In this analysis, each event is considered a "node", with orientation as node 1 and predation as node 2. The presence or absence of each event was analyzed as binary data with a binomial distribution (López‐Sepulcre et al., 2015). In analysis of the data from the experiment with *T*. *castaneum* from the stock culture, the starvation condition of *A*. *venator* was considered a fixed effect and the ID of the *A*. *venator* was considered a random effect. In analysis of the data from the experiment using the strains of *T*. *castaneum* selected for duration of death feigning, the starvation condition of the *A*. *venator* and strains of the beetles were considered fixed effects, and the ID of the *A*. *venator* was considered a random effect. In analysis of the data from the experiment using the strains of *T*. *confusum* selected for duration of death feigning, the strains of the beetles were considered fixed effects and the ID of the *A*. *venator* and replicate lines were considered random effects.

We also tested orientation and predation separately in a logistic regression analysis as a post‐hoc test of the item responses tree models. In analysis of the data of experiment with *T*. *castaneum* from stock culture, we considered the starvation duration, sex of *A*. *venator*, and interaction between these factors fixed effects, and the day of the experiment a random effect. In analysis of the data of experiments with beetles from death‐feigning strains, the starvation duration, sex of *A*. *venator*, strains of *T*. *castaneum*, and interactions among these factors were considered fixed effects, and the day of the experiment was considered a random effect. In the analysis of data of experiment with beetles from death‐feigning strains, the starvation duration, sex of *A*. *venator*, the strains of *T*. *confusum*, and interactions among these factors were considered fixed effects, and replicate lines of death‐feigning strains [lines A–C] and the experimental day were considered random effects.

All statistical analyses were performed using R ver. 3.4.3 (R Core Team, 2017) and used programs of *lme4* (Bates et al., 2015) and *car* (Fox & Weisberg, 2019) in these analyses.

## 3 RESULTS

### 3.1 Experiment 1: Freezing behavior

**FIGURE 3:**
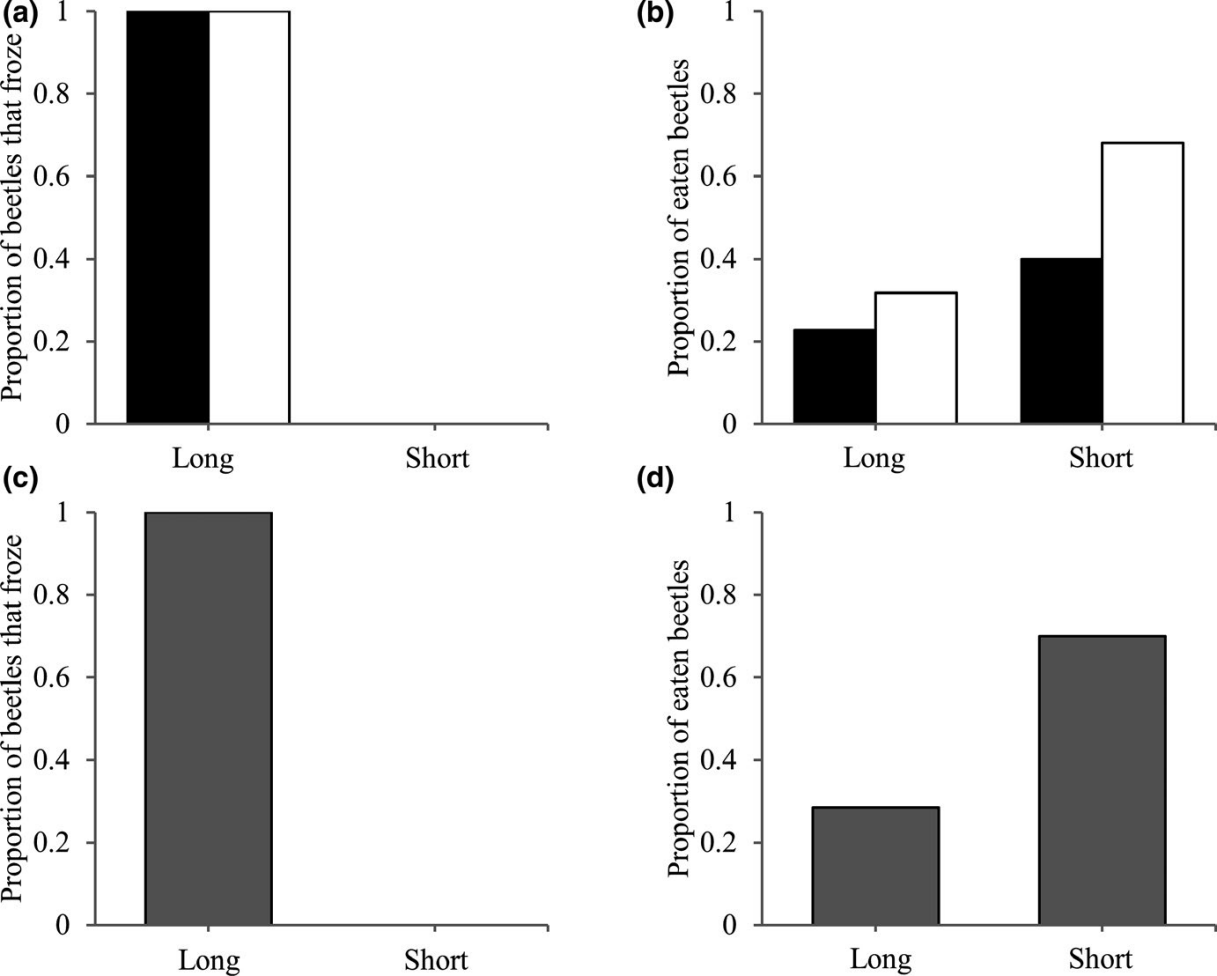
Proportions of freezing (a and c) and being preyed on (b and d) of beetles from long (L) and short (S) strains of *Tribolium castaneum* (a and b) and *T*. *confusum* (c and d) in encounters with non‐starved *Amphibolus venator*. Black and white bars show males and females of *T*. *castaneum*, respectively, and gray bars show sexually unclassified adults of *T*. *confusum*

Figure 3 shows results of experiments focused on the freezing of *T*. *castaneum* and *T*. *confusum*. In both *T*. *castaneum* (Figure 3a) and *T*. *confusum* (Figure 3c), 100% of individuals in the long strains froze, but no individual in the short strains did, and the frequency of freezing behavior highly significantly differed between the strains. About 60% of the short‐strain beetles of *T*. *castaneum* were preyed on, while about 30% of the long‐strain beetles were preyed on (Figure 3b). About 70% of the short‐strain beetles of *T*. *confusum* were preyed on, while about 30% of the long‐strain beetles were preyed on (Figure 3d).

Table 1 shows results of item response tree analyses for data obtained by the experiments. In both beetle species, there are significant effects of interaction between node and strain, indicating that freezing behavior of the beetles affects the predation behavior of *A*. *venator* (Tables 1 and 2). Generalized linear mixed‐model (GLMM) results for predation in both beetle species indicated that beetles from short strains showed a significantly lower freezing rate (Figure 3a, c, Tables 1 and 2) and a higher predation rate than long strains (Figure 3b, d, Tables 1 and 2).

**TABLE 1:** Item responses for tree analyses of data by experiments focused on freezing of beetles

Beetle	Factor	df	*χ* ^2^	*p*
*T. castaneum*	Node	2	2.25	.3251
	Node*strain	2	6.51	.**0387**
	Error	167		
*T. confusum*	Node	2	0.02	.9903
	Node*strain	2	7.19	.**0275**
	Error	75		

Bold values are statistical significances in *p* values.

**TABLE 2:** GLMM for proportions of freezing and predation on *T*. *castaneum* from long and short strains on encountering a non‐starved predator. In results of freezing of *T*. *castaneum*, all beetles showed freezing

Beetle	Trait	Factor	df	*χ* ^2^	*p*
*T. castaneum*	Freezing	Strain	1	0	1
		Sex	1	0	1
		Strain*sex	1	0	1
		Error	82		
	Predation	Strain	1	6.42	**.0113**
		Sex	1	3.16	.0757
		Strain*sex	1	0.56	.4533
		Error	82		
*T. confusum*	Freezing	Strain	1	56.81	**<.0001**
		Error	39		
	Predation	Strain	1	7.25	**.0071**
		Error	39		

Bold values are statistical significances in *p* values.

### 3.2 Experiment 2: Effect of starvation period and orientation behavior of predator

Table 3 shows results of item response tree analyses. In the results of beetles from the stock culture, there was a significant effect of interaction between node and starvation treatment of *A*. *venator*, indicating that starvation treatment affects the predation behavior of this bug (Table 3). In results of both death‐feigning strains *T*. *castaneum* and *T*. *confusum*, there were significant effects of interaction between node and strain, indicating that the death‐feigning behavior of the beetles affects the predation behavior of *A*. *venator* (Table 3).

**TABLE 3:** Item responses for tree analyses of data obtained by experiments with beetles from stock culture of *T*. *castaneum*, death‐feigning strains of *T*. *castaneum*, and death‐feigning strains of *T*. *confusum*, respectively

Beetle	Factor	df	*χ* ^2^	*p*
Stock culture	Node	2	76.25	**<.0001**
	Node*starvation	4	10.12	.**0385**
	Error	253		
*T. castaneum*	Node	2	3.61	.1646
	Node*starvation	2	2.56	.2784
	Node*strain	2	33.39	**<.0001**
	Node*starvation*strain	2	0.32	.8512
	Error	138		
*T. confusum*	Node	2	109.85	**<.0001**
	Node*strain	2	48.30	**<.0001**
	Error	658		

Bold values are statistical significances in *p* values.

**FIGURE 4:**
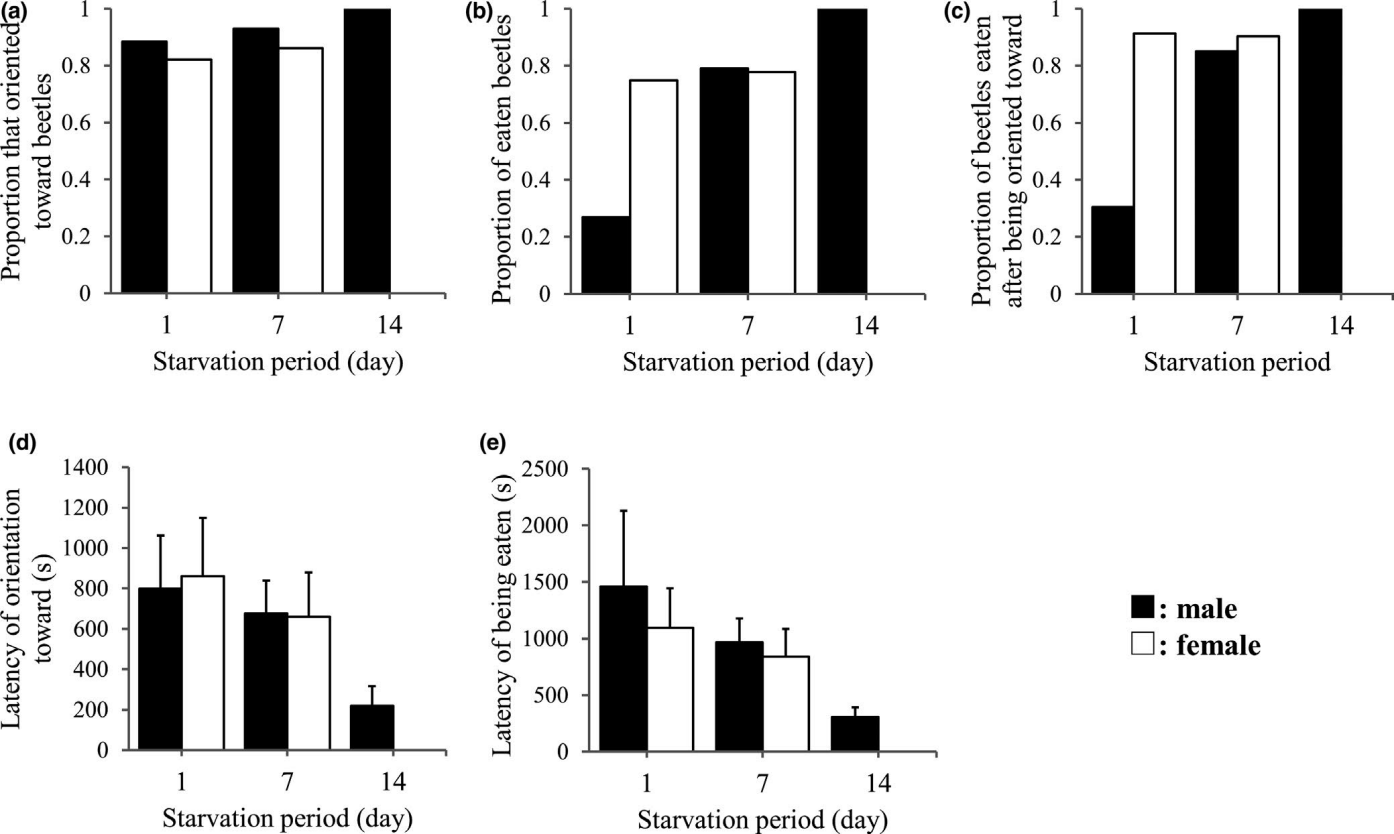
Effects of starvation periods (1, 7, and 14 days) of predator on (a) proportion of orientation toward beetles, (b) proportion of preyed‐on beetles, (c) proportion of preyed‐on beetles after orientation toward, (d) latency of orientation toward beetles, and (e) latency of being preyed of beetles in predation experiments using *Tribolium castaneum* as prey and *Amphibolus venator* as predator. Black and white bars show males and females of *A*. *venator*, respectively. Error bars shows standard error

Figure 4 shows the effects of starvation duration on predation with *T*. *castaneum* from the stock culture. Table 4 shows results of GLMM for orientation rate, latency of orientation, predation rate, latency of predation, and predation after orientation in experiments with beetles from the stock culture (=base population). There were no significant effects of starvation treatment, sex, or their interaction in the orientation rate (Figure 4a, Table 4). In results of predation rate, there was a significant effect of sex, indicating that females of *A*. *venator* had a significantly higher predation rate than males (Figure 4b). There were no significant effects of starvation treatment and interaction between starvation treatment and sex in the predation rate (Table 4). Similarly, there was a significant effect of sex in the predation rate after orientation, indicating that females of *A*. *venator* experienced a significantly higher predation rate after orientation than males (Figure 4c, Table 4). There was also a significant effect of interaction between starvation treatment and sex in this result, but not starvation treatment alone (Table 4). In the latencies of orientation and predation, although Figure 4 shows decreases of these latencies with starvation periods (Figure 4d, e), there were no significant effects of the starvation treatments and other factors (Table 4).

**TABLE 4:** GLMM to test effects of starvation of *A*. *venator* on orienting toward and predation of *T*. *castaneum* from stock culture population

Trait	Factor	df	*χ* ^2^	*p*
Orientation rate	Starvation	2	0.48	.7884
	Sex	1	1.55	.2133
	Starvation*sex	1	0.04	.849
	Error	133		
Latency of orientation	Starvation	2	1.18	.5531
	Sex	1	0.01	.9182
	Starvation*sex	1	0.1	.7484
	Error	117		
Predation rate	Starvation	2	4.49	.1061
	Sex	1	3.97	.**0463**
	Starvation*sex	1	10.03	.0015
	Error	133		
Latency of predation	Starvation	2	1.73	.4212
	Sex	1	0.59	.4426
	Starvation*sex	1	0.03	.8688
	Error	90		
Predation after orientation	Starvation	2	4.32	.1153
	Sex	1	5	.**0253**
	Starvation*sex	1	5.88	.**0153**
	Error	117		

Bold values are statistical significances in *p* values.

**FIGURE 5:**
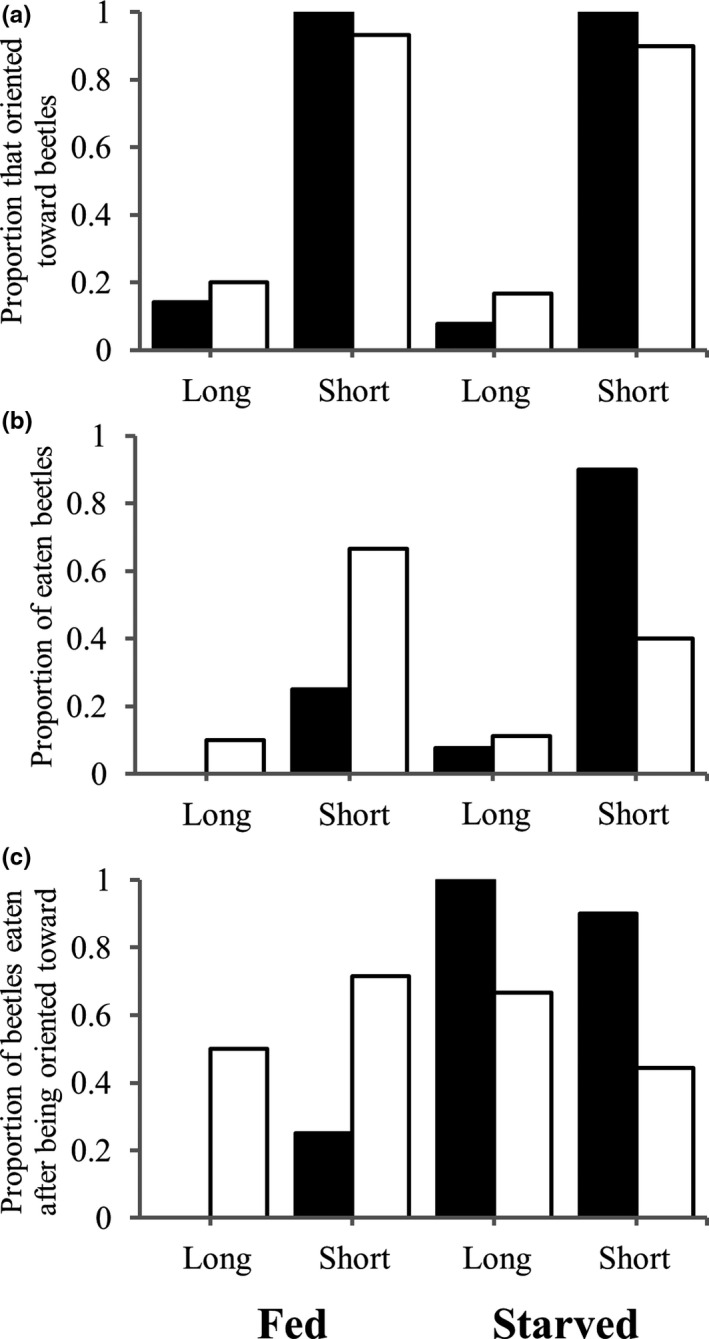
Results of predation experiments when short and long strains of *Tribolium castaneum* encounter “Fed” and “Starved” (7d) *Amphibolus venator*. Graphs show (a) proportions of predators orientated toward beetles, (b) proportion of preyed on beetles, and (c) proportion of beetles that were preyed on after being oriented by a predator. Black and white bars show male and female *A*. *venator*, respectively. L and S show long and short strains, respectively

Figure 5 shows results of predation experiments with *T*. *castaneum* from death‐feigning strains. Table 5 shows results of GLMM for data of this experiment. In results of orientation rate, there was a significant effect of strain (Table 5), indicating that beetles from short strains showed a significantly higher rate of being orientated toward *A*. *venator* than long strains (Figure 5a). There were no significant effects of starvation treatment, sex, and their interactions (Figure 5a, Table 5). Similarly, beetles from short strains were preyed on at a significantly higher rate by *A*. *venator* than long strain (Figure 5b, Table 5). A significant effect was also found in the interaction between starvation treatment and sex (Table 5), indicating that starved males showed higher predation rates than females (Figure 5b). Other factors did not indicate a significant effect on predation rate (Table 5). In results of predation rate after orientation, there was a significant effect of interaction between starvation treatment and sex (Table 5), indicating that starved males showed a higher predation rate after orientation than females regardless of beetle strain (Figure 5c). Other factors did not have a significant effect on this result.

**TABLE 5:** Results of GLMM to test effects of starvation of *A*. *venator* on orienting toward and predation of *T*. *castaneum* from death‐feigning strains

Trait	Factor	df	*χ* ^2^	*p*
Orientation rate	Strain	1	19.13	**<.0001**
	Starvation	1	0.28	.5952
	Sex	1	0.54	.462
	Strain*starvation	1	0.01	.9028
	Strain*sex	1	0	1
	Starvation*sex	1	0.07	.7951
	Strain*starvation*sex	1	0	1
	Error	87		
Predation rate	Strain	1	16	**<.0001**
	Starvation	1	0.18	.6746
	Sex	1	0.38	.54
	Strain*starvation	1	0.64	.423
	Strain*sex	1	2.94	.0867
	Starvation*sex	1	8.64	.**0033**
	Strain*starvation*sex	0		
	Error	88		
Predation after orientation	Strain	1	0.01	.9342
	Starvation	1	0.37	.5454
	Sex	1	0.51	.4744
	Strain*starvation	1	0.78	.3768
	Strain*sex	1	0	.9749
	Starvation*sex	1	8.54	.**0034**
	Strain*starvation*sex	1	0	.9632
	Error	44		

Bold values are statistical significances in *p* values.

**FIGURE 6:**
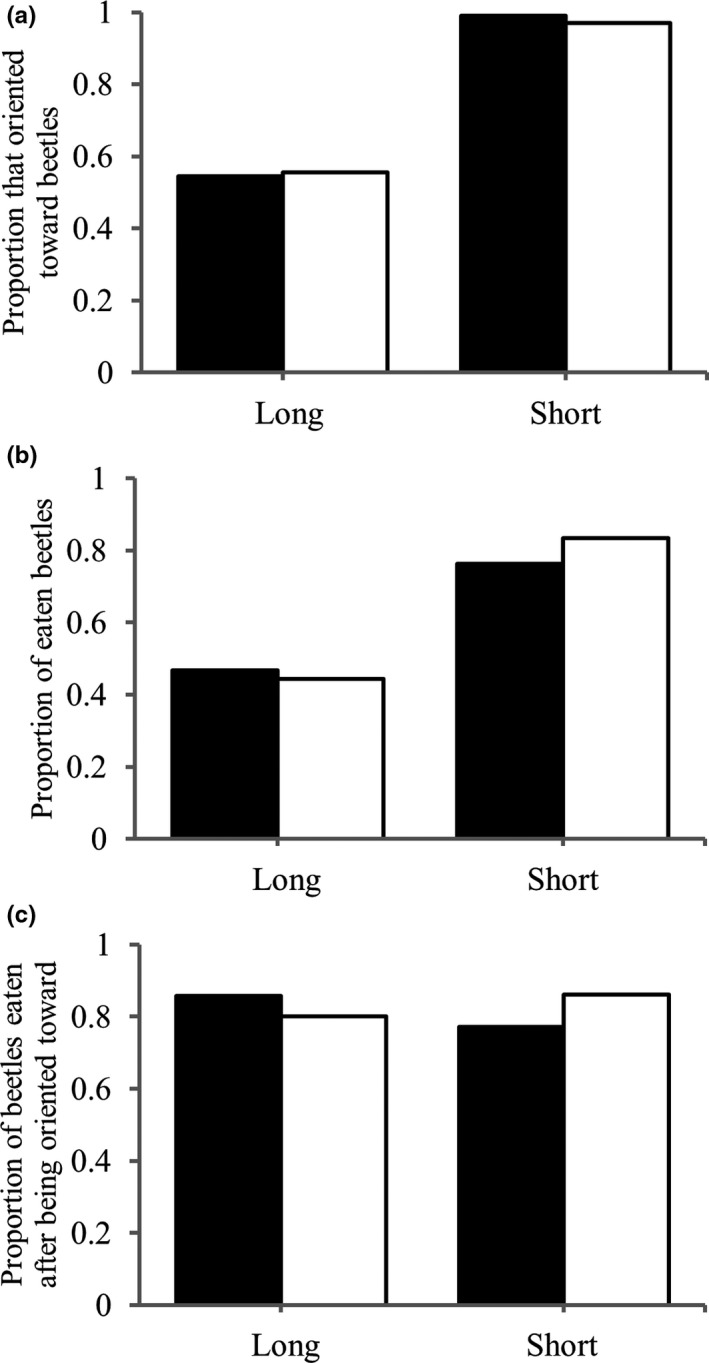
Results of predation experiments in which *Tribolium confusum* encountered with fed and starved (7d) *Amphibolus venator*. Graphs show (a) proportions of predators oriented toward beetles, (b) proportion of preyed‐on beetles, and (c) the proportion of beetles that were preyed on after being orientated toward a predator. Black and white bars show males and females of *A*. *venator*, respectively. L and S show long and short strains, respectively

Figure 6 shows results of predation experiments with *T*. *confusum* from long and short strains. Table 6 shows results of GLMM for data obtained by this experiment. In results of orientation rate, there was a significant effect of strain (Table 6), indicating that *A*. *venator* more frequently oriented toward beetles from the short strain than the long strain (Figure 6a). There were no significant effects of sex and interaction between strain and sex in orientation rate (Table 6). Similarly, the predators preyed significantly more on beetles from short strains than long strains (Figure 6b, Table 6). There were no significant differences in the effects of sex and interaction between strain and sex in predation rate (Table 6). In the results of predation rate after orientation, there were no significant effects of all factors (Figure 6c, Table 6).

**TABLE 6:** Results of GLMM to test effects of starvation of *A*. *venator* on foraging for *T*. *confusum* from death‐feigning strains

Trait	Factor	df	*χ* ^2^	*p*
Orientation rate	Strain	1	41.49	**<.0001**
	Sex	1	0.01	.9195
	Strain*sex	1	0.94	.3317
	Error	370		
Predation rate	Strain	1	21.49	**<.0001**
	Sex	1	0.31	.5772
	Strain*sex	1	1.51	.219
	Error	370		
Predation after orientation	Strain	1	0.03	.8632
	Sex	1	0.52	.4706
	Strain*sex	1	3.41	.0647
	Error	285		

Bold values are statistical significances in *p* values.

## 4 DISCUSSION

Previous studies have shown that when long‐strain *Tribolium* beetles are attacked by the pursuit‐type predator, *H*. *adansoni*, they exhibited death‐feigning behavior to survive (Matsumura, Yumise, et al., 2021; Miyatake et al., 2004, 2009; Nakayama & Miyatake, 2010). In contrast, the results of the present study showed that *Tribolium* beetles did not exhibit death‐feigning behavior against a sit‐and‐wait type predator, *A*. *venator*, but froze instead.

Freezing is defined as stopping moving when a prey senses the presence of an enemy, and is different from death feigning (Sakai, 2021). The conventional view of immobility shows that the same prey will initially “freeze” in a series of behavioral processes against the predator, and if the predator still does not give up, it will move to “death feigning” as a last resort (Hennig, 1979; Honma, 2021; Humphreys & Ruxton, 2018; Sakai, 2021). However, the present results showed that *Tribolium* beetles exhibited different antipredator strategies, freezing, or death feigning against different natural enemies. This may provide one answer to the ecological mystery of how predators evolve antipredator strategies for multiple enemies in nature (Hoverman & Relyea, 2007; Sih et al., 1998).

Interestingly, the beetle strains of *T*. *castaneum* and *T*. *confusum* selected for short duration of death feigning did not exhibit freezing but simply ran away. In contrast, the long‐strain beetles of both species, which were selected for long duration of death feigning, did not use death feigning in a series of steps against *A*. *venator* in the present study, although they used death feigning against *H*. *adansoni* (Miyatake et al., 2004). This meant that *Tribolium* beetles have the ability to use different tactics depending on the enemy they meet.

The fact that the long‐strain beetles showed different predator‐avoidance tactics against different types of predator is a new finding that surprised us. This difference in antipredator tactics may be due to the fact that *H*. *adansoni* is generally a pursuit‐type predator, and contact stimuli result in death‐feigning behavior, whereas *A*. *venator* is a sit‐and‐wait type predator (Matsumura, Yumise, et al., 2021), and only moderate contact with beetles occurs, and thus beetles never feign death (see Video 2). With a sit‐and‐wait predator, the tactic of freezing until the enemy walks away unnoticed will be adaptive because this type of predator is not interested in prey unless it is moving (see Video 2). It will be interesting to examine how *Tribolium* species detect the species of different types of enemies by more detailed behavioral observation in the future. Additional research is needed to investigate what predator avoidance strategies organisms adopt in response to contactless stimuli by predators in various taxa. For example, a recent study has shown that the Italian wall lizard, *Podarcis siculus*, shows death‐feigning behavior even in the absence of direct contact (Damas‐Moreira, 2021).

The long‐strain beetles that feign death suffered a lower predation rate than short‐strain beetles that did not feign death. However, this study showed that even in the long‐strain beetles, once oriented toward the predator, were preyed upon with the same high frequency as in the short‐strain beetles in both species *T*. *castaneum* and *T*. *confusum*. This suggests that being detected and orientated toward the movement of the prey is fatal to the prey species.

The results also suggested that freezing was effective against a non‐starved *A*. *venator* but may not be effective against a starving *A*. *venator*. The fact that the starvation level of a predator alters the efficacy of possible antipredator tactics taken by the prey will provide a novel perspective for research of antipredator behaviors (McNamara, 1987; Quinn et al., 2012).

In a previous study, the short‐strain *T*. *freemani* beetles with higher activity were eaten by *A*. *venator* significantly more often than the long‐strain beetles with lower activity (Konishi et al., 2020). In Konishi et al. (2020), latencies of predation and survival rates during 5 min of beetles with *A*. *venator* were compared between the strains. What is the reason for the high frequency of predation on the short‐strain beetles, with essentially little death feigning toward *T*. *freemani*, when the present study showed *Tribolium* species do not feign death against *A*. *venator*?

One possible answer may lie in the genetic link between death‐feigning behavior and activity. In previous experiments, the short‐strain beetles walked longer distances than the long‐strain beetles in all four beetle species: *T*. *castaneum* (Miyatake et al., 2008), *T*. *confusum* (Nakayama et al., 2010), *T*. *freemani* (Konishi et al., 2020), and *Callosobruchus chinensis* (Nakayama & Miyatake, 2008). The results of a study using *T*. *castaneum* indicate that dopamine expression in the brain is involved in this correlation of the two behaviors, death feigning, and locomotor activity (Nishi et al., 2010). The expression of dopamine in the brain is significantly higher in the short strains than in the long strains (Miyatake et al., 2008). Molecular‐level comparisons using RNAseq have also shown that the expression of dopamine‐related genes differs between the strains (Tanaka et al., 2021; Uchiyama et al., 2019).

Therefore, it is reasonable to assume that the results of a previous experiment (Konishi et al., 2020), in which the strains with a shorter duration of death feigning were preyed upon more frequently than the strains with a longer duration of death feigning are related not to the effect of death feigning, but the genetic link between death feigning and walking activity. In other words, short strains with high dopamine expression in their brains, which show less death feigning and usually move around a lot, are more likely to be found by natural enemies because they move around more than usual. The results of the present study also showed that *A*. *venator* more frequently oriented toward the short‐strain beetles than toward the long‐strain beetles. And that led to a high predation rate. The high prey availability of the short‐strain beetles is offset by the adaptive benefits of frequent encounters with potential mates (Nakayama & Miyatake, 2010). We, thus, consider that the relationship between dopamine‐activity and death‐feigning duration at genetic levels evolved variations in the duration of death feigning in natural populations in *T*. *castaneum* (Konishi et al., 2020). It makes logical sense that individuals with shorter and longer durations of death feigning can coexist in the field. The present results suggest that the high frequency of predation on *T*. *freemani* (Konishi et al., 2020) was not due to the effect of death feigning, but rather because the short strain, which shows less death feigning, has instead higher walking activity than the long‐strain beetles (Konishi et al., 2020). The present results suggest that this is because the short strain, which shows less death feigning, has higher walking activity than the long‐strain beetles (Konishi et al., 2020) and is more frequently detected, oriented toward, and then preyed upon by predators.

In a study using a locomotor activity detector with *T*. *castaneum*, we subjected the long strain, which was selected for long walking distances, and the short strain, which was selected for short walking distances, to predation by *A*. *venator*, and the short‐strain beetles were preyed upon significantly more often than the long‐strain beetles (Matsumura & Miyatake, 2015).

The presence of predatory assassin bugs, such as *A*. *venator*, and jumping spiders as natural enemies is universally observed, especially in subtropical regions, in the flour mills where *Tribolium* beetles live. Therefore, examining the relationship between the densities of predatory bugs and spiders, and antipredator behaviors of prey in the field will provide an important ecological perspective for research on avoiding attack, that is, on the level of population and community ecology. On the other hand, if we establish an experimental model in which two predator species and one prey species live together in a room to test multiple predator effects, we can develop research on the question of how multiple predator strategies with molecular links based on dopamine (death feigning, freezing, or escaping) make phenotypic changes against multiple predators in relation to ecological conditions (see Nakayama et al., 2012).

In conclusion, the two behaviors, freezing and death feigning can be better put in the context of proximate causes of the behavioral switch against different predators underlying a pleiotropy between the two antipredator behaviors that are mediated by dopamine, at least in the present case.

## CONFLICT OF INTEREST

All authors have no competing Interest.

## AUTHOR CONTRIBUTIONS

**Masaya Asakura:** Data curation (lead); Writing – original draft (equal). **Kentarou Matsumura:** Formal analysis (supporting); Visualization (equal). **Ryo Ishihara:** Data curation (equal). **Takahisa Miyatake:** Conceptualization (lead); Formal analysis (supporting); Funding acquisition (equal); Investigation (lead); Methodology (lead); Project administration (lead); Supervision (lead); Visualization (equal); Writing – original draft (lead); Writing – review & editing (lead).

## Supporting information

Appendix S1

Appendix S2

## Data Availability

https://datadryad.org/stash/share/2wlwnAp60sfJiMJotIGwPp0Dlwuhu2WZNzObGgYzqWw. https://doi.org/10.5061/dryad.djh9w0w0x_v2.
