# Supplementary material for: Freezing or death feigning? Beetles selected for long death feigning showed different tactics against different predators
Source: Ecol Evol. 2022 Feb 7;12(2):e8533. doi: 10.1002/ece3.8533 (PMC8820118; doi:10.1002/ece3.8533)
Supplement: Supplementary file 2 — Appendix S2 [file ECE3-12-e8533-s002.docx]

**Supplementary information**

**Table S1** Sample size of *Amphibolus venator*, *Tribolium castaneum*, and *T. confusum* in each experiment.

**Table S2** AIC values of each distribution in analyses of latency of orientation and latency of predation, respectively.

**Table S1**. Sample size of *Amphibolus venator*, *Tribolium castaneum*, and *T. confusum* in each experiment.

| *A. venator* | Starva-tion period (day) | Sample size of *T. castaneum* | | |  | Sample size of *T. confusum* | |
| --- | --- | --- | --- | --- | --- | --- | --- |
|  |  | Stock culture | Death feigning strain | |  | Death feigning strain | |
|  |  |  | L-strain | S-strain |  | L-strain | S-strain |
| Experiment 1*^1^ |  |  |  |  |  |  |  |
| Male | 7 |  | 22 | 22 |  |  |  |
| Female | 7 |  | 22 | 20 |  |  |  |
| Unclassified | 7 |  |  |  |  | 21 | 20 |
|  |  |  |  |  |  |  |  |
| Experiment 2^*2^ |  |  |  |  |  |  |  |
| Male | 1 | 26 | 7 | 12 |  |  |  |
|  | 7 | 43 | 13 | 10 |  | 90 | 97 |
|  | 14 | 5 |  |  |  |  |  |
| Female | 7 | 28 | 10 | 15 |  |  |  |
|  | 7 | 36 | 18 | 10 |  | 90 | 97 |

*1: Predation experiment focused on freeze behavior of beetles.

*2: Predation experiment focused on orientation and predation rate of predators.

L-strain and S-strain showed long and short strains, respectively.

**Table S2.** AIC values of each distribution in analyses of latency of orientation and latency of predation, respectively.

| Trait | AIC | | |
| --- | --- | --- | --- |
|  | Gamma | Gaussian | Poisson |
| Latency of orientation | 1829.9 | 2017.5 | 104127.2 |
| Latency of predation | 1490.5 | 1581.2 | 84866.8 |
